# Quantifying the visual salience of Swedish vowel contrasts: a computer vision approach

**DOI:** 10.3389/fpsyg.2026.1826567

**Published:** 2026-09-04

**Authors:** Helene Springer, Henrik Garde, Frida Splendido, Marianne Gullberg

**Affiliations:** 1 Centre for Languages and Literature, Lund University, Lund, Sweden; 2 Lund University Humanities Lab, Lund, Sweden

**Keywords:** computer vision, lip configurations, Swedish vowel contrasts, visual salience, visual speech signal

## Abstract

Seeing a speaker’s face can facilitate speech comprehension, but the magnitude of this effect depends on the visual salience of speech sounds. Visual salience has traditionally been defined through descriptions of speakers’ articulatory gestures using discrete binary features. We examined which contrasts among Swedish long vowels are visually different and whether these align with traditional binary feature descriptions based on vowel openness and lip rounding. This proof-of-concept case study quantifies lip configurations in continuous speech to characterize the visual information available to listeners. Using a computer-vision approach, we extracted the area, height, and width of the mouth opening from video-recorded Swedish speech produced by a single speaker. Despite gradual variation within the vowel categories, the results reveal visually distinguishable groups of vowels. Crucially, they suggest that production-based binary feature matrices can misrepresent the visual information available to addressees, particularly in continuous speech. We discuss the advantages of quantitative visual parameters over traditional discrete features based on tongue position and lip rounding for describing visual speech. We also evaluate the use of computer vision for extracting facial landmarks and measuring lip configurations.

## 1 Introduction

Visual information from lip movements has been demonstrated to be beneficial for speech recognition since the mouth and lips provide cues on the segmental and suprasegmental level. However, in order to examine how the visual signal of speech is perceived, a description of the visual signal is required. These visual descriptions are not as thoroughly and systematically made as the descriptions for their acoustic counterparts in the auditory signal. Descriptions are often based on binary, articulatory features (i.e., from the speaker’s perspective), or on phonological features (i.e., from a theoretical perspective), and it has not yet been examined whether those descriptions necessarily align with the gradual variations of the visual parameters available in naturalistic audiovisual speech. Moreover, the prediction of potential audiovisual speech benefits in human vowel perception still relies heavily on descriptions reduced to the presence or absence of openness and roundedness, or speechreading studies presenting isolated or sustained vowels. Therefore, the current study aims to characterize the geometric properties of the lip configurations of Swedish vowels to examine visual vowel contrasts in continuous speech.

Speech perception is a multimodal process in which visual signals from the face are constantly incorporated to form a perceptual outcome. McGurk and MacDonald (1976) demonstrated the powerful influence of the visual signal on perception by presenting multimodal mismatches and showing that listeners perceive the sound of the syllable *ba* over a silent video of a speaker saying *ga* as a fused percept *da*. Studies comparing auditory with audiovisual listening conditions (i.e., seeing the speaker’s face) have found that comprehension of speech is facilitated, when the speaker’s face is visible and the acoustic and visual signal of speech align naturally. This is referred to as an audiovisual benefit, which applies both to speech perception in noise or with competing speakers (Ross et al., 2007; Jesse and Janse, 2012), and to clear listening conditions, when listening is rendered more challenging due to semantic complexity, syntactic structure, or accent (Arnold and Hill, 2001). Another line of study focuses on differences between listening in a first or second language (L1 and L2, respectively), and effects of proficiency in the target language. Findings show that facilitating effects of audiovisual speech exist in both L1 and L2 listening, despite a tendency for L1 listeners to outperform L2 listeners (Fitzpatrick and Kim, 2010; Drijvers and Özyürek, 2020). On the segmental level of speech, the facilitating effects are largest for visually high-salient sounds or sound contrasts, which is reflected both in behavioral and neurophysiological measures (Hazan et al., 2005; van Wassenhove et al., 2005; Jesse and Janse, 2012). For example, an event-related-potential (ERP) study investigated the auditory-evoked N1 and P2 component during audiovisually presented speech and showed that temporal facilitation is greater for high-salient bilabial consonants than for low-salient velar consonants (van Wassenhove et al., 2005).

Vowel contrasts in audiovisual speech can be described from a theoretical perspective (i.e., using phonological features), from the speaker’s perspective (i.e., describing articulatory processes), and from the addressee’s perspective (i.e., describing the acoustic and visual cues that may be perceived and decoded by the addressee). Most of the theoretical and articulatory descriptions are based on discrete features, ranging from binary to three- or four-way distinctions, whereas acoustic and visual cues relevant for perception can also be described as gradient parameters.

Auditory contrasts of vowel quality, for example, can be described in terms of phonological features, including openness, backness, and roundedness. These are traditionally based on the articulators, such as tongue position or the rounding of the lips (Lindau, 1978). In other words, these descriptions are based on the speaker’s articulatory gestures. In the acoustic speech signal, vowel quality contrasts are typically examined for three parameters: The first formant (F1), which is inversely related to openness, the difference between the second and the first formant (F2–F1) which is directly related to tongue backness, and a lowering of the second and third formant (F2 and F3) indicating lip rounding (Ladefoged and Johnson, 2010). From the addressee’s perspective, acoustic differences in vowel contrasts (and hence, their potential perceptual salience) can therefore be described on a continuous scale and can be measured directly from the speech signal.

In a number of studies on audiovisual speech perception, vowel contrasts are described as (putatively) visually salient when they differ in the phonological features openness and roundedness (Robert-Ribes et al., 1998; Traunmüller and Öhrström, 2007; Inceoglu, 2022). These features are considered to be more salient in visual perception because their correlates in the visual signal are on the lips and the mouth, whereas other features such as the backness of the tongue are less salient simply because they cannot be seen.

Beyond descriptions of the presence or absence of speaker-based phonological features in the visual signal, there are a number of studies focusing on speechreading performances. So-called visemes (visual phonemes), groups of vowels and consonants that can be perceived as visually contrastive, are established in these studies by measuring confusion rates (Fisher, 1968). Such an addressee-based description of visual similarity of Swedish vowels has been provided in two speechreading studies. Amcoff (1970) listed three groups of lip configurations for long vowels in Swedish (/iː, eː, ɛː/, /ɒː, øː, yː/, and /oː, ʉː, uː/), whereas for short vowels, the distinction was made mainly between rounded and unrounded vowels. Mártony (1974) presented three groups /iː, eː, ɛː/, /ɒː/, and /øː, yː, oː, ʉː, uː/, also suggesting that the distinctive feature in the visual signal was vowel openness and roundedness. Henceforth, we will further refer to abstract groups and meaning-distinctive phoneme categories of a vowel with the // notation (e.g., the vowel phoneme /ɒː/), the [] notation indicates a concrete realization of the vowel sound, or a measured vowel item (e.g., the measured vowel item [ɒː]).

From a perceptual perspective, there are several drawbacks of describing the visual signal by means of speechreading performances or phonological features. First, basing a visual description on speechreading performances risks introducing variations due to differences in lipreading abilities. While this risk can be mitigated by testing multiple speechreaders in a repeated-measures design, the existing speechreading studies examine visual discriminability of Swedish vowels only in isolated words, which may not necessarily reflect the speechreading performance for vowels in continuous speech. Second, the use of binary phonological features risks neglecting fine-grained, gradual differences in continuous speech, for example in roundedness and openness. In fact, a substantial body of research has demonstrated that audiovisual speech contains a considerable amount of individual speaker variation affecting both acoustic and visual realizations of vowels (Johnson et al., 1993; Bear and Harvey, 2017). This poses challenges for both human and machine perception of audiovisual speech. Variation also appears to be related to the linguistic context in which vowels occur in a continuous speech signal (Cole et al., 2010), which can also affect the lip configurations that appear in the signal. While Automatic Speech Recognition (ASR) and lipreading systems already are trained on continuous speech data, we propose that continuous speech data should also be considered when describing speech contrasts for research purposes.

Finally, the binary features can clearly be misleading concerning the actual visual properties of speech. If a simple binary account of openness and roundedness is used to describe the visual signal from the addressee’s perspective, the Swedish vowel /yː/ will be characterized as more similar to /ʉː/ (both are –open, +rounded, +front) than to /iː/ (**−**open, **−**rounded, +front). However, the visual speech signal suggests that /ʉː/ could be the vowel phoneme that stands out due to its narrow mouth opening, as seen in Table 1. The close front part of the Swedish vowel space is particularly interesting, since the acoustic distances between the vowels /iː/, /ʉː/ and /yː/ are rather small, and perceptually challenging even for L1 listeners (Gross and Forsberg, 2020). The question, then, is whether visual information enhances the perceptual contrast. As Gross and Forsberg (2020) suggest, the case of /yː/ and /iː/ may be an early-stage merger with lip rounding as a perceptually weak feature. While a binary phonological feature description can account for the visual differences in some languages, the example in Table 1 shows that treating roundedness as a binary feature is not sufficiently informative for Swedish, which has two different types of lip rounding. *Compressed rounding* appears when the lips converge vertically which leads to a narrow mouth opening as for /ʉː/, whereas *protruded rounding* is an articulatory gesture which involves moving the lips forward laterally, as for /yː/ (Lindau, 1978). These differences related to the different rounding gestures may be better captured with quantified descriptions of lip configurations than by ascribing potential perceptual salience effects to the absence of a roundedness or openness feature. Measures of the visual parameters from continuous speech provide a quantified baseline of the visual signal, which may allow for more accurate predictions about the salience of the visual information that can be used in audiovisual speech perception, particularly in languages with dense vowel systems.

**Table 1:** Phonological feature descriptions of the Swedish long vowel phonemes /iː/, /yː/, and /ʉː/, and their visual appearance.

	Phoneme /iː/	Phoneme /yː/	Phoneme /ʉː/
± open	–	–	–
± rounded	–	+	+
± front	+	+	+
Visual similarity	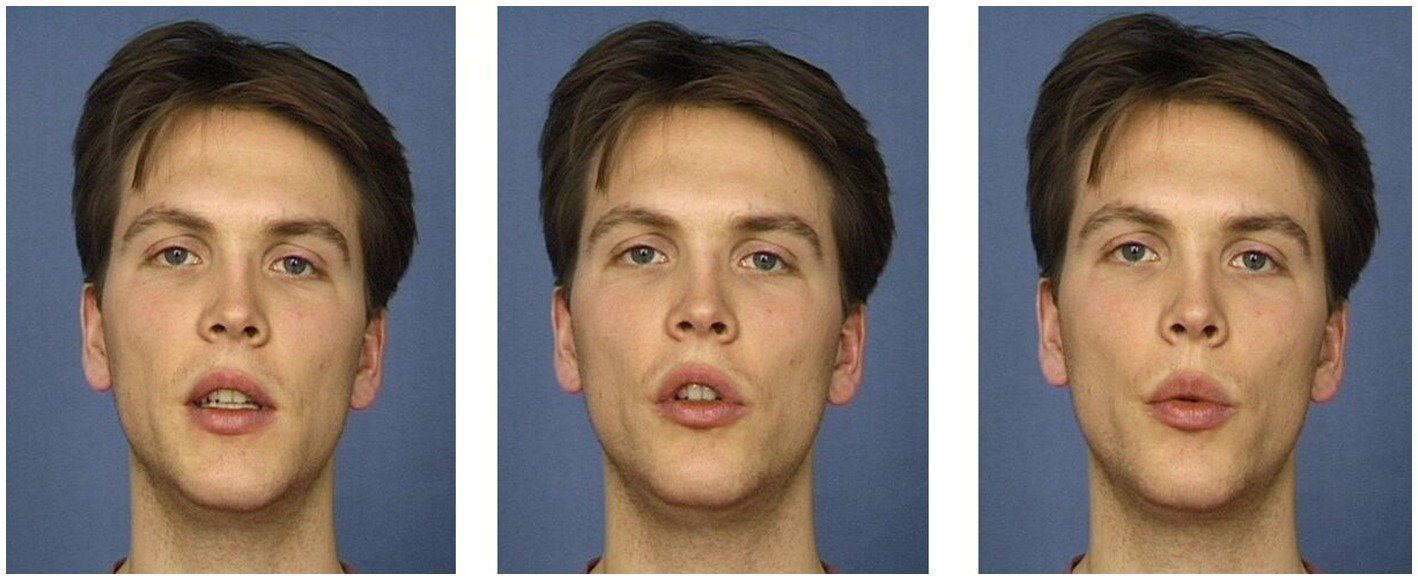		

Phonological feature accounts differ in terms of the backness feature, some accounts state that /ʉː/ is a central vowel. However, in strictly binary descriptions, it is classified as +front.

The way the acoustic and visual speech signal is described has implications for the concept of perceptual salience, not only concerning experimental designs but also for the way human perception is characterized. Although the perceptual outcome in speech perception often involves a decision for a specific category, it is likely that the underlying perceptual encoding is sensitive to continuous information in the speech signal (McMurray, 2022). Therefore, quantified descriptions of the visual parameters related to openness and roundedness could act as reference points for describing the visual differences between vowels that may be relevant for perception, similar to formant frequency scales in acoustic descriptions.

As a matter of course, the visual signal includes several parameters that can indicate openness and lip rounding in the two- and three-dimensional plane. In Fromkin’s (1964) simple account of English vowel lip configurations, the measures of the vertical opening of the mouth (height) and the horizontal spread of the corners of the mouth (width) were highlighted as more informative than lip protrusion and area of the mouth opening, since the latter two can be predicted from the height and width (see also Abry and Boë, 1986). These parameters may not distinguish specific vowel phonemes from one another, but they can be sufficient to establish visually contrastive groups (Fromkin, 1964). For Swedish vowel quality, Linker (1982) also named lip protrusion as a crucial parameter.

Several methods have been employed previously to quantify area, height, width, and other visual parameters of speech sounds from different angles, focusing on both lip and tongue configurations. Earlier approaches have made use of photographs from both lateral and frontal perspectives (Fromkin, 1964; Linker, 1982), and x-ray imaging or stone casts for three-dimensional measurements (Fromkin, 1964). Other studies have used video recordings and MR data (Ericsdotter, 2005), 3D electromagnetic articulography (Garnier et al., 2012), optical motion capture (Nordstrand et al., 2004; Green et al., 2010), and the blue-lip technique to extract visual parameters of lip configurations (Garnier et al., 2010; Lo et al., 2023).

As one of the more recent methods to extract visual parameters, computer vision approaches have considerable advantages for multimodal perception research compared to the above-mentioned methods, for example the possibility of using already existing video or picture material to extract the measurements efficiently. Krause et al. (2020) measured vertical lip aperture by using landmarks in the OpenFace 2.0 face tracking system, and demonstrated that this approach was suitable for investigating lip aperture contrasts (see also Krause and Kawamoto (2020) for measures of horizontal aperture).

Dlib and MediaPipe are two well-known libraries for facial landmark tracking. Their face detection scores and accuracy have been tested and compared in a number of studies (Savin et al., 2021; Wojnar et al., 2023). Application areas of these libraries are predominantly emotion recognition (Wojnar et al., 2023), person identification (Rasanayagam et al., 2018), automated proctoring of students during online exams (Maniar et al., 2021), recognizing drowsiness in vehicle operators (Albadawi et al., 2023), medical contexts including diagnostic purposes (Fan et al., 2022) and patient monitoring (Roesler et al., 2022), and the development of systems for automatic cued speech recognition (Sankar et al., 2022). However, none of these tests focuses specifically on the mouth area or on the accuracy of speech measurements for the investigation of visual vowel contrasts.

**Figure 1:**
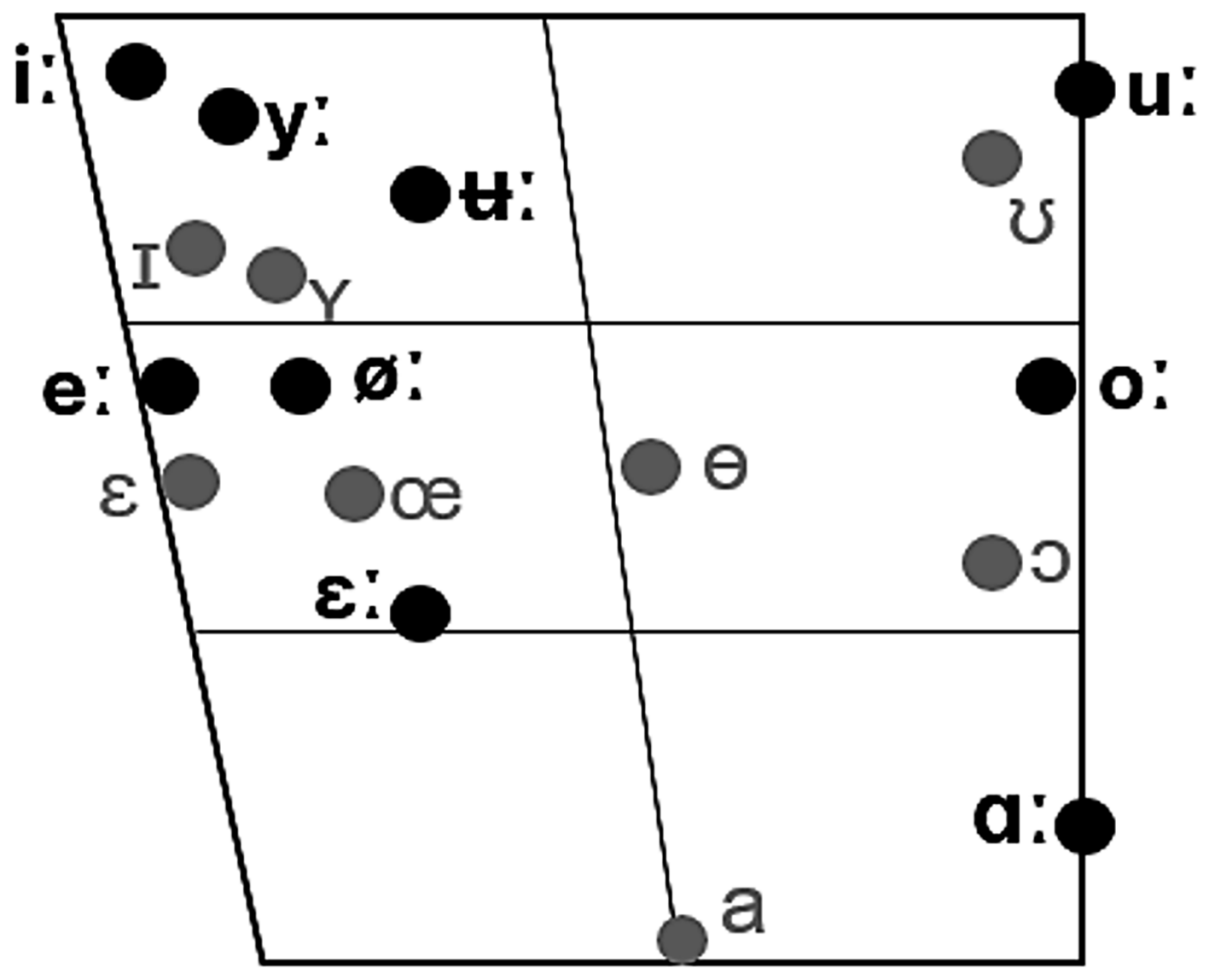
Vowel diagram of the Swedish vowel system (after Engstrand, 1999). Long vowels are displayed with black circles and short vowels are displayed with grey circles.

The aim of the current study is to characterize lip configurations in Swedish long vowel contrasts as being of high or low visual salience as they appear in the visual signal and as they may be perceived by addressees. The Swedish vowel system contains nine long vowel categories (displayed in Figure 1). The geometric properties of the mouth opening form the basis of our analysis. Taking articulatory gestures of vowels as a starting point, the openness feature may be quantified as the inner area of the mouth opening since the mouth opening is typically larger in open vowels such as /ɛː/ than in closed vowels such as /iː/, because the tongue position is lower in +open vowels than in –open vowels. The opening gesture may also lead to an increased height for +open vowels. In other words, moving the tongue upward toward the palate in close vowel articulation brings the lips closer together, decreasing both area and height. The roundedness feature can be quantified as the width of the mouth opening, which should be smaller for all the vowels phonologically classified as +rounded in both compressed and protruded rounding, although the different types of lip rounding may differ in width. Two previous studies (Linker, 1982; Ericsdotter, 2005) have examined the properties of lip configurations to distinguish vowels in Swedish, including measures of the parameters area, height and width, embedded in different carrier syllables or words, or recorded as individual sustained vowels. The current case study focuses on the visual lip configurations of vowels embedded in *continuous speech* produced by one speaker.

These geometric properties of the lip configurations are then related to the articulatory descriptions that are prevalent in the literature. This is particularly important in light of multimodal perception research claiming to focus on the addressee’s perspective, yet using predominantly speaker-based descriptions in categorizing vowels as high- and low-salient in perception. Table 2 displays the nine vowel categories and their binary feature specification.^1^ Note that the matrices in Table 2 are reduced to the features considered to be crucial for visual contrast. In the full featural description by Riad (2013), for example, the front-back dimension is specified as more fine-grained by including three levels (front-central-back), and the height dimension is specified by four levels (high, mid-high, mid, low). These dimensions are not considered in the current study because they describe the tongue position, which is visually not accessible. However, these features reflect internal articulatory differences and therefore, are crucial for any phonological feature account uniquely specifying each vowel category. Therefore, the frontness feature would fully specify the difference between /ɒː/ and /ɛː/, as well as /oː, uː/, /ʉː/, and /yː, øː/. The height feature would further specify differences between /oː/ and /uː/, and /yː/ and /øː/, respectively. Moreover, the speechreading evidence by Amcoff (1970) and Mártony (1974) indicates that visual differences probably appear between /ɒː/ and /ɛː/ as well, presumably due to rounding, and that /ɛː/ may show more visual similarities with /eː, iː/. However, it remains an open question whether feature-based and speechreading-based descriptions are in line with visual measurements of Swedish vowels in continuous speech.

**Table 2:** Phonological binary feature matrix for the long vowels of Swedish and for the features openness and roundedness, based on Engstrand (1999).

Phonological feature	/ɒː/	/ɛː/	/eː/	/iː/	/oː/	/uː/	/ʉː/	/yː/	/øː/
Open	+	+	−	−	−	−	−	−	−
Rounded	−	−	−	−	+	+	+	+	+

To test this, we measure the parameters area, height, and width in the complete set of long Swedish monophthongs, thus enabling a systematic comparison of the vowels in comparable, yet naturalistic contexts to answer the following questions:

*RQ_1_*: Which Swedish vowel contrasts show significant differences in the visual signal in terms of area, height, and width of the mouth opening in continuous speech?

*RQ_2_*: Do the binary phonological descriptions of vowel roundedness and openness match the visual lip configurations of Swedish vowels?

We define a matching description as a lack of significant difference between the vowels whose binary phonological feature matrices are identical in terms of openness and roundedness. We hypothesize that vowel contrasts with identical feature matrices for openness and roundedness (Table 2) do not show significant differences in the visual parameters area, height, and width, and conversely, vowel contrasts with differences in feature matrices for openness and roundedness (Table 2) show significant differences in at least one of the visual parameters area, height, and width. Although we expect gradual differences within vowel categories (i.e., for different realizations of the same phoneme) and between vowels with identical feature matrices for openness and roundedness, we expect these to be less pronounced and to lack significance.

## 2 Materials and methods

**Figure 2:**
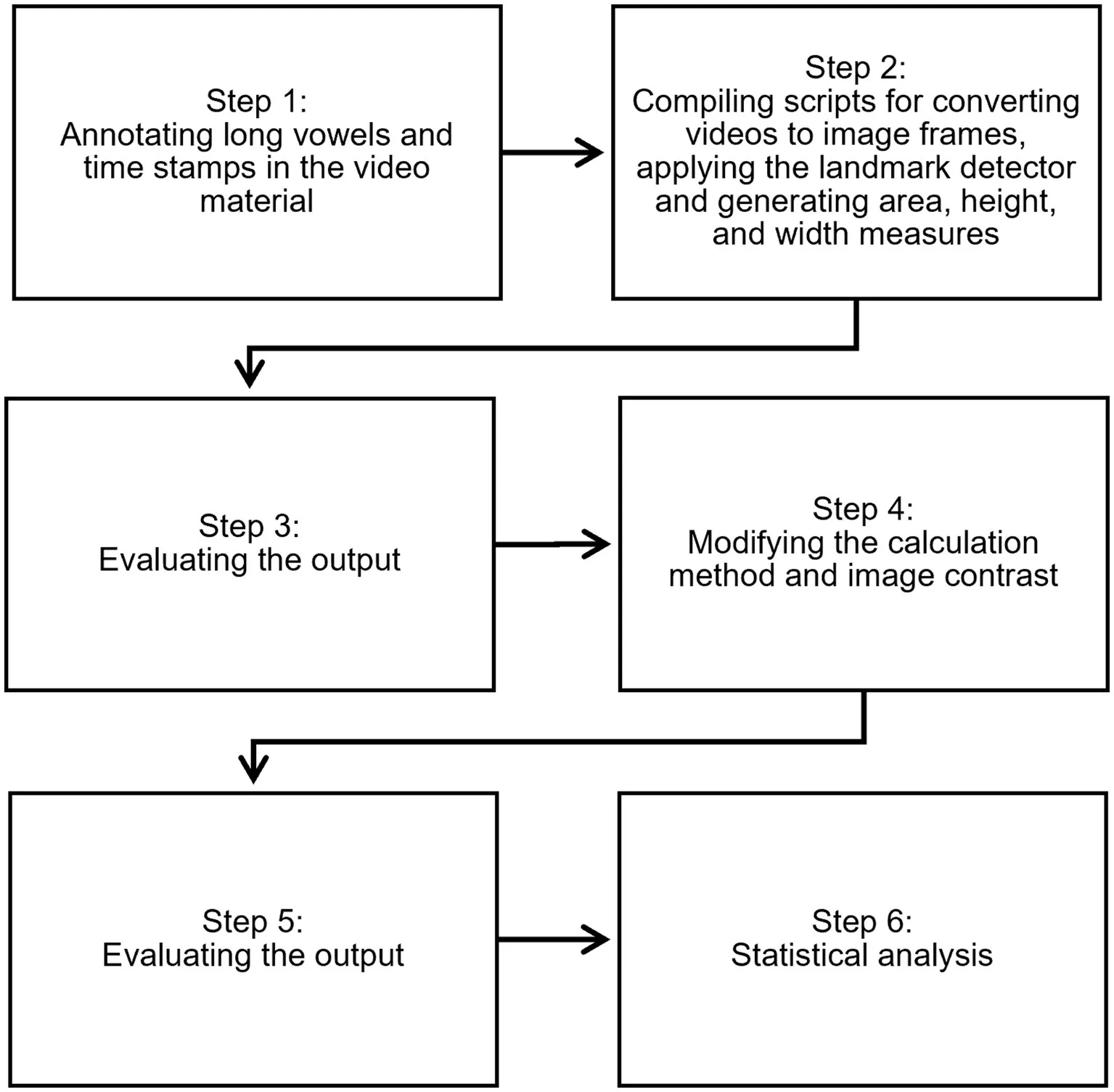
Method flow chart. The numbers in the boxes indicate the section in which the respective methodological step is explained in this paper. The arrows indicate our procedure. At each evaluation point, previous steps can be iterated, modified, and repeatedly evaluated.

Figure 2 provides an overview of the procedure. First, the vowel items were annotated from the video material. Second, several scripts were compiled that split videos into image frames and detected the outline of the mouth opening on these frames, and returned area, height, and width. After a first evaluation, the scripts were further refined, and the image frames were edited in order to improve the measurements. After an additional evaluation, the provided measures were analyzed statistically. Below, the methods are presented in considerable detail in order to introduce the method and facilitate its development for future work. At each evaluation point, a previous methodological step can be iterated, modified, and evaluated again.

### 2.1 Preparation and annotation of the visual material (step 1)

The measurements are based on an audiovisual database (Öhman, 1998), consisting of 180 short sentences with an average duration of 2.3 s. The sentences have simple syntactic structures and simple semantic contexts and were spoken by one male speaker of Standard Central Swedish (Stockholm area) in a neutral speaking style with typical reductions and coarticulations. The speaker was guided by synthesized speech to keep the speech rate constant. Highlights and shadows in the video were not reduced in a systematic manner; however, a light source in the form of a light bulb of 500 W and a semi-transparent umbrella were part of the setting (Öhman, 1998). The stimuli were recorded from a frontal perspective with a Sony DCR-VX1000E digital camera, and for the audio recordings, a microphone was positioned on the speaker’s lapel. Öhman (1998) compressed and corrected the recorded video files to ensure synchrony between audio and video track. The files have a frame size of 440 × 333 pixels.

From this video database, 213 long vowels were selected. The long vowels /æː/ and /œː/ occurred in the material as the pre-r allophones of /øː/ and /ɛː/. These allophones are characterized by articulatory-acoustic differences (Schumacher, 2014). Therefore, they need to be considered separately from /øː/ and /ɛː/. However, due to the number of items (*n* = 7 for /æː/, and *n* = 1 for /œː/), they were excluded from the analysis, and a sample of 205 items remained for the analysis. Table 3 shows the distribution of items across vowel categories. The vowel category /ɒː/ occurred most often in the sample (*n* = 52), while the categories /ɛː/, /øː/, /yː/ were represented by only 11, 11 and 6 items, respectively. Only long vowels were chosen because they have stable measurable midpoints. Although speech sounds unfold and change dynamically in continuous speech as the speaker forms an utterance (coarticulation), midpoint static moments from long monophthongs were chosen as key moments for the measurements. However, the method can be extended to measure multiple frames per vowel token to account for coarticulatory phenomena. In order to be included in the sample, vowels had to be embedded in a CVC speech context to ensure none of the vowel tokens was adjacent to another vowel.

**Table 3:** Distribution of vowel items in the annotated dataset.

Vowel	/ɒː/	/ɛː/	/eː/	/iː/	/oː/	/uː/	/ʉː/	/yː/	/øː/
Frequency	52 (0.25)	11 (0.05)	28 (0.14)	20 (0.1)	25 (0.12)	31 (0.15)	21 (0.1)	6 (0.03)	11 (0.05)

Absolute frequencies and proportions in parentheses.

The time stamps of the midpoints of these vowels were annotated in the video annotation software ELAN (version 5.3; Wittenburg et al., 2006) and used to identify the corresponding video frames of the vowel items. The places of articulation for adjacent consonant phonemes were documented. In Swedish, long vowels can only occur in stressed syllables (Riad, 2013). None of the items was therefore subject to reduction. Since the overall number of items were rather small, sentence-initial (*n* = 36) and sentence-final (*n* = 12) syllable vowels were included in the dataset, if they were not the sentence-initial or sentence-final *phoneme* of the sentence. In cases of perceptual ambiguity, vowels in sentence-final syllables were inspected in Praat (version 6.1.04; Boersma, 2001) to ensure that none of these items were subject to reduction and that all items met the vowel length and stress requirements.

### 2.2 Implementing the landmark detectors (step 2)

**Figure 3:**
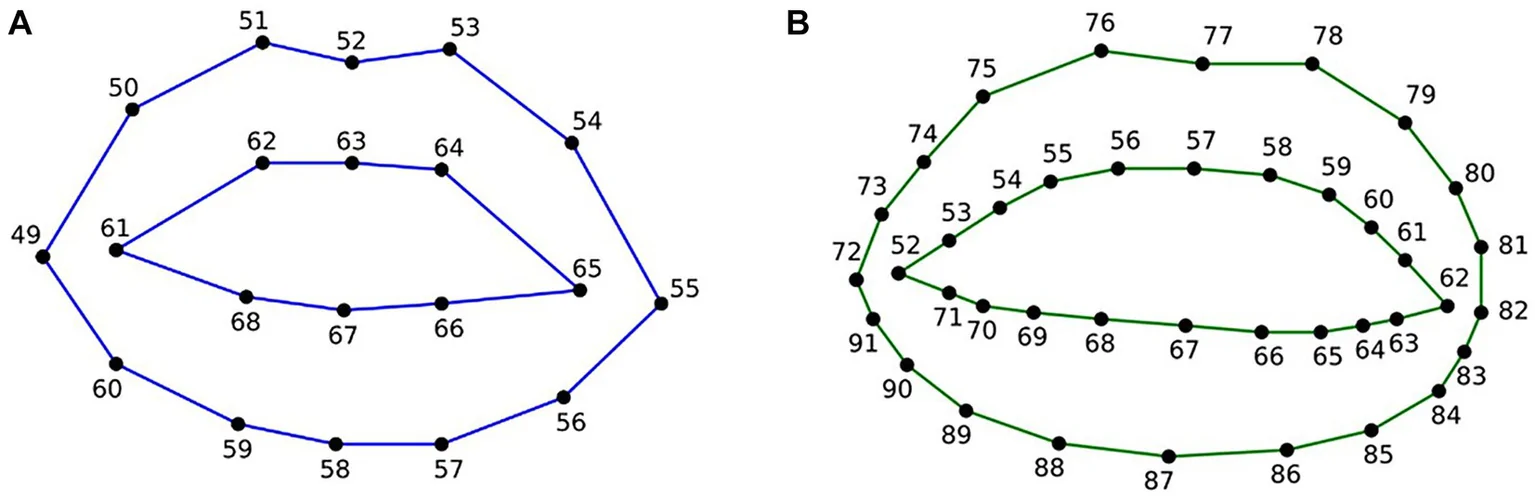
Facial landmark models based on Dlib **(A)** and MediaPipe (**B**, simplified version). Displayed are the mouth regions only, the original models include landmarks for the entire face.

To extract the measurements of area, height, and width of the mouth opening from the videos, two Python scripts were customized that incorporate each of the two computer vision libraries Dlib (version 19.24.2; King, 2009) and Google’s MediaPipe (version 0.10.0; Lugaresi et al., 2019). Figure 3 shows the mouth region of the two facial landmark models that these libraries are based on. Dlib’s model is based on eight landmarks for the inner outline and 12 for the outer outline, and MediaPipe’s model tracks the inner mouth outline with 20 landmarks, and 20 landmarks tracking the outer outline (Figure 3). However, logic indices were specified in the beginning of the script and only the corresponding eight landmarks of MediaPipe were used to facilitate comparison with the Dlib output. Although jaw movements and lip protrusion can be informative for visual vowel perception (Fromkin, 1964), the frontal perspective of the video material and the non-fixated head of the speaker allowed us to measure the area, height, and width of the mouth opening, establishing a focus on two-dimensional measures of the mouth and lips.

The complex workflow was split into several steps. Each step executed an OS command or Python script that produced output in the form of folders, text, and images, including intermediate results that served as input for the next step and to assess the functioning of the method. The workflow consisted of a sequence of steps that could be executed and re-executed one by one and for a single video file, but the entire sequence could also be executed for a batch file containing a large amount of video files.

First, the script split the video material into picture frames in bmp format with a frame rate of 40 milliseconds. The time stamps of the vowels were matched to the closest video frame following the annotated time stamp. In the second step, the landmarks of Dlib and MediaPipe were detected on these frames, and the coordinates of the mouth in the (x,y) plane were saved in a text file. These coordinates were later used to calculate the area in pixels within a polygon that connects the landmarks of the mouth opening. A polygon is a closed, geometrical figure connecting several points in a two-dimensional plane. In this case, we connected the points of the landmarks along the inner mouth outline to achieve a figure that reflects the area of the mouth opening. Horizontal (width) and vertical (height) measures of the mouth opening in pixels were calculated by counting the difference between the maximum and minimum value on the x- and y-axis, respectively (subtraction method). The last step in the batch summed up the result in a single file. The detailed documentation and information about the script is available at the Open Science Framework.^2^

### 2.3 Evaluation of the first script output (step 3)

In this step, we identified aspects to be improved for the final measurements. First, we checked whether the frames assigned to the vowel items corresponded to the intended vowels. Three items were corrected manually. Second, the polygons were visually inspected to check whether the intended landmarks were accurately placed on the face. Third, the numerical output in the output files was evaluated.

**Figure 4:**
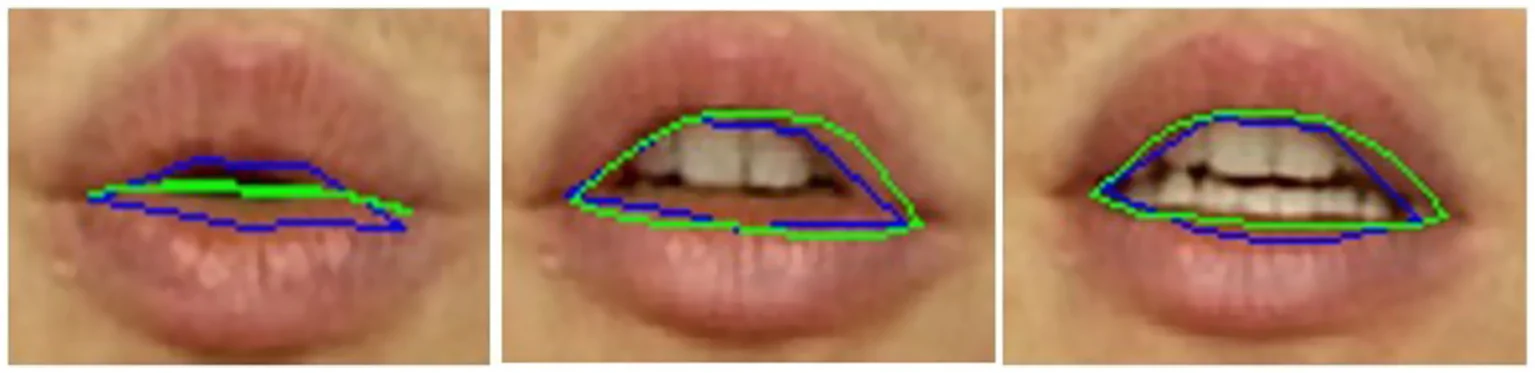
Examples of output polygons for the vowels [oː], [øː] and [ɛː] (from left to right). Dlib’s polygons are displayed in blue, MediaPipe’s polygons in green.

Figure 4 shows examples of three vowel tokens [oː], [øː] and [ɛː] and the detected polygons on the images frames. The visual inspection of both Dlib’s and MediaPipe’s polygons showed that the landmarks on the corners of the mouth were often located incorrectly, and polygons tended to be too large rather than too small (Figure 4). A general tendency of MediaPipe’s landmarks was to be more accurate on the lower lip, and less precise on the upper lip contour, whereas Dlib’s landmarks showed the opposite pattern (Figure 4, right panel). Another notable pattern was the frequent misplacement of the landmarks on the lower lip contour, most likely due to the light reflections on the lower lip.

**Figure 5:**
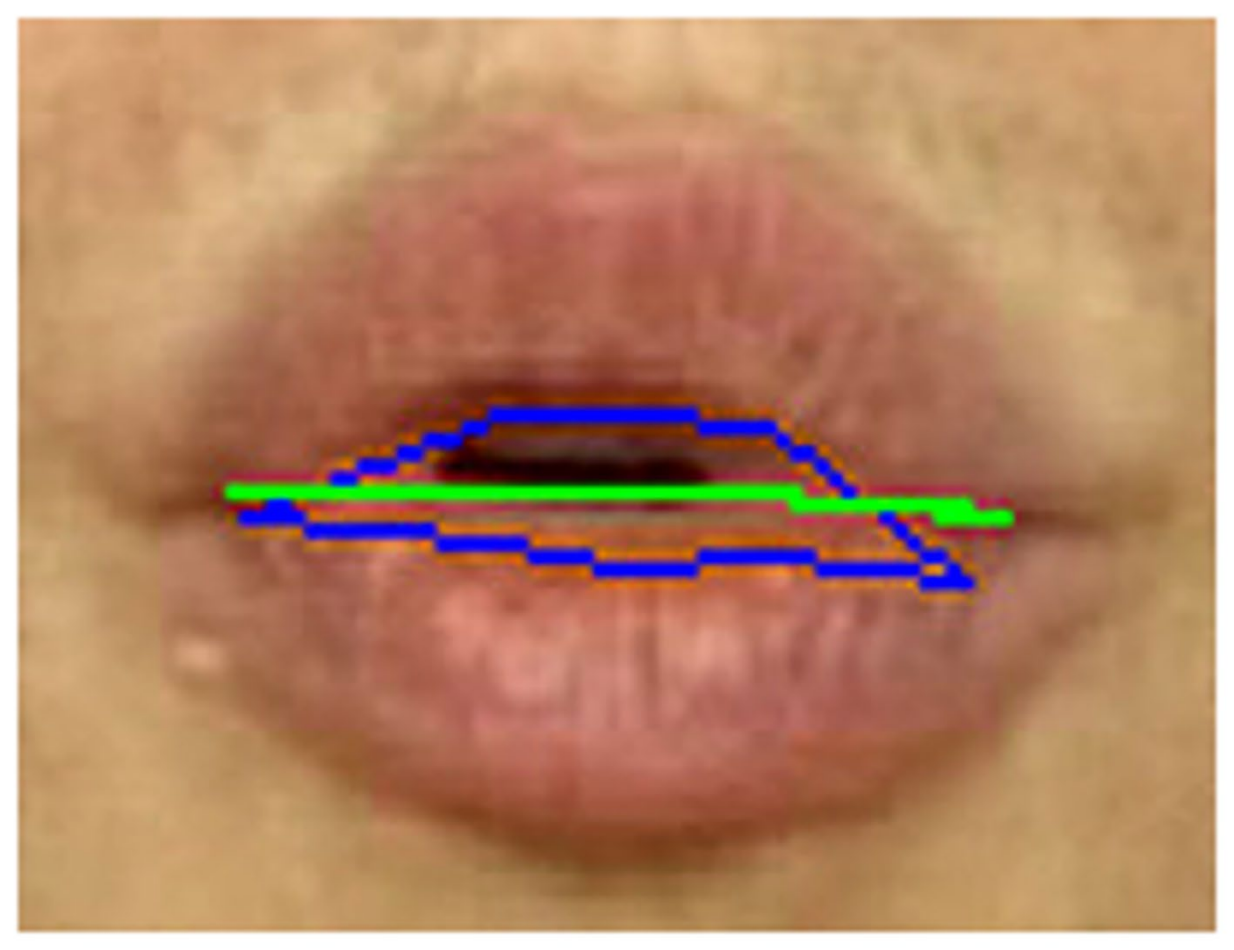
Example of polygon output for the vowel [uː]. Dlib’s polygons are displayed in blue, MediaPipe’s polygons in green.

For vowels with a narrower mouth opening (/ʉː/, /oː/, and /uː/), MediaPipe failed to detect an opening at all in 40 cases. This is illustrated in Figure 5, which shows the image frame for the target vowel token [uː] and a green line showing that MediaPipe did not detect a mouth opening. MediaPipe further tended to approximate the polygon shape to a symmetric shape, whereas Dlib seemed to recognize asymmetries better. For both libraries, the speaker’s left corner of the mouth was often substantially misplaced, almost converging with the outer corner of the mouth, or pulled down toward the darkest pixels in the mouth area. The oral commissures of the bottom and upper lip created a shadow that may have been misinterpreted as the inner corner of the mouth, most likely due to the algorithms’ sensitivity to the contrast between lighter and darker pixels. Moreover, for rounded vowels, there might not be a distinct corner of the mouth. Instead, the rounding smoothed the edge of the corner and made it more difficult to detect.

The numerical output of the script was checked in two ways. First, the text files were examined. These did not always provide the exact difference between maximal and minimal x and y values. There was a margin of approximately 1–2 pixels in the checked items in terms of the height and width parameters. However, the overall numerical values still seemed reasonable considering the polygons. Second, the image analysis software Fiji (with the underlying architecture of ImageJ, version 1.54f) (Schindelin et al., 2012) was used to check numerical values in the output files for area, height, and width measurements that deviated strongly from the other data points in the same vowel category. ImageJ includes image analysis tools such as the wand tracing tool, the polygon selection tool, and straight, segmented, or freehand lines, and it includes measuring and analyzing functions providing area and length data in pixels. It served as a manual control tool for the measurements provided by the script, although it only could determine the approximate accuracy of the measurements. While the Dlib measurements from our script matched the manually performed control measurements approximately, a number of MediaPipe measurements from the script did not align with the manually measured polygons. For instance, there were height values close to 0 (i.e., the non-existent polygons), and in other cases, the numerical value did not match the manual control measures as closely as Dlib’s values did. Note that there was a visible mouth opening on all the measured frames, even where MediaPipe failed to recognize an opening and interpreted it as a straight line.

### 2.4 Modifications (step 4)

**Figure 6:**
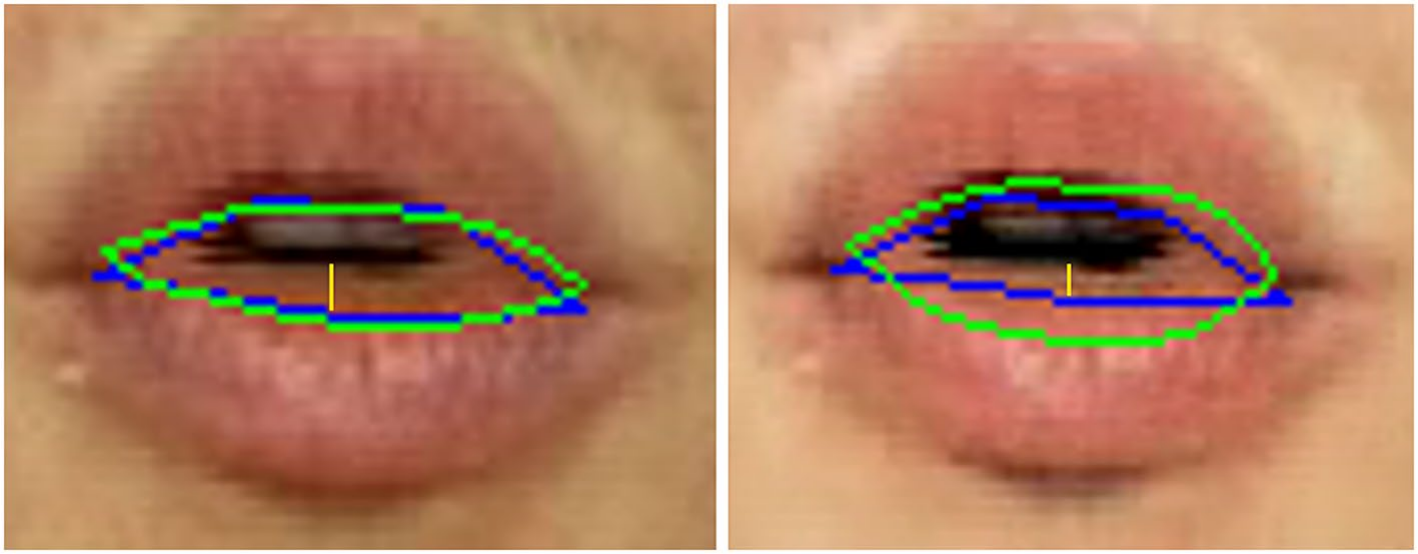
Manual control measurements. The yellow vertical line illustrates the distance between the actual and the detected mouth opening for Dlib in the contrast frames (right) as compared to the raw frames (left). Dlib’s polygons are displayed in blue, MediaPipe’s polygons in green.

Since the initial inspection of the output suggested effects of lighting conditions, visual effects were manipulated on one of the video stimulus sentences to increase the contrast between lips, teeth, and mouth opening, thereby improving the accuracy of landmark detection. Monochrome effects made the detection of mouth outlines less accurate by obliterating differences between the colors and textures of the different tissues and structures of the mouth area, including lips, teeth, and skin. However, enhancing the contrast of the raw video material using the software Adobe Premiere Pro (effect Contrast, level 150) increased the contrast in these areas which made the location of the landmarks more accurate, especially on the central part of the lower lip. In Figure 6, the manual control measurements are illustrated by displaying raw and contrast versions of an image frame and a detected mouth opening side by side, illustrating to what extent the polygons deviated from the actual mouth opening, and how this deviance could be reduced in the Contrast150 version (henceforth referred to as the *contrast* video, the original material henceforth referred to as *raw* video).

**Figure 7:**
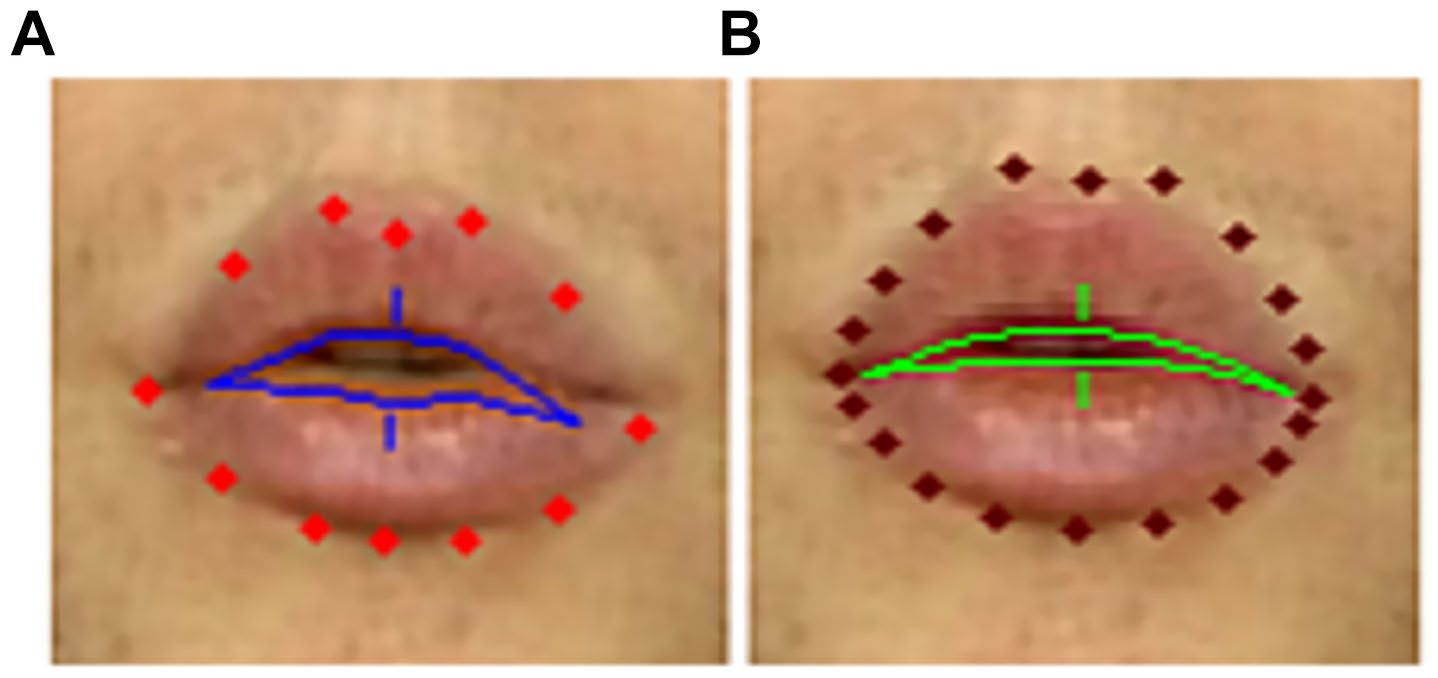
Output frames including the polygons tracing the landmarks along the inner mouth opening. Vertical lines indicate the central landmark positions used for the height calculations, and additional landmarks for the outer mouth outline for Dlib (blue, left panel, **A**) and MediaPipe (green, right panel, **B**).

The subtraction method required a certain degree of symmetry in the polygons. As Figures 4, 5 show, this was not present for all items. To improve the measurements in this regard, height and width were calculated differently, by using the distance between the coordinates of the specific landmarks (Dlib height: y_landmark 63_ and y_landmark 67_, Dlib width: x_landmark 61_ and x_landmark 65_, MediaPipe height: y_landmark 57_ and y_landmark 67_, MediaPipe width: x_landmark 52_ and x_landmark 62_). Figure 7 shows the central landmark positions for two vowel tokens through vertical lines and further illustrates that the most dominant error source was the speaker’s left corner of the mouth (i.e., to the right in the image). However, the points in Figure 7 tracing the outer mouth outline show that this was not due to the libraries’ algorithm confusing inner and outer mouth outlines. We expected this misplacement to persist in the contrast videos, and that the width measurements would not improve to the same extent by the central landmark calculations as the height measurements.

### 2.5 Evaluation of the final script output (step 5)

The final output consisted of area, height, and width measurements for Dlib’s and MediaPipe’s landmarks in both the raw and the contrast videos. Along with the measures of the subtraction method, central measures of height and width, and additional annotations of the analyzed vowels, we compiled a database of visual lip configurations (see text footnote 1). The aim of the final evaluation step was to establish which of these measures was best suited to answer the research question about the visual differences between the lip configurations in Swedish vowels.

The measurements selected were related to the features openness and roundedness, which made the detection of a mouth opening an essential aspect required from the script output. The deviating polygons were annotated, and Dlib’s algorithm had a success rate of 100% (*n* = 205) in detecting a mouth opening in the raw and contrast videos, whereas MediaPipe showed notable difficulties in detecting a mouth opening in both video versions (80%, *n* = 165 in the raw videos, and 87%, *n* = 179 in the contrast videos). Since MediaPipe’s measurements would lead to the exclusion of at least 26 items to avoid a severe error source of failed polygon detections, the final analysis was based on Dlib’s measurements.

In the next step, Dlib’s polygons were visually inspected and evaluated for accuracy by two raters, comparing the number of outliers of the raw videos and the contrast videos. If one or both of the landmarks on the corners of the mouth (landmark 61 and 65) were misplaced or inaccurate, they were marked as outliers for the width parameter. If one or both of the landmarks on the central upper and central lower lip were misplaced or inaccurate (landmark 63 and 67), the item was marked as an outlier in the height dimension. This manual annotation was hampered by the poor resolution in the zoomed-in frames, in which the boundaries between teeth and lips were blurred, which particularly affected the annotation of the height parameter. In the contrast videos, these boundaries were often easier to detect, but ambiguities still occurred. In general, the tendency remained for the configurations of the inner mouth shape of vowels with a larger opening to be more accurately detected than vowels with a narrow opening. Interrater reliability agreement was assessed using Cohen’s Kappa on 213 items. Cohen’s kappa values indicate a slight agreement for the height parameter in the raw video frames (*κ* = 0.09, *p* = 0.014), fair agreement for the height parameter in the contrast video frames (*κ* = 0.27, *p* < 0.001), and substantial agreement for the width parameter in both the raw (*κ* = 0.71, *p* < 0.001) and contrast video frames (*κ* = 0.73, *p* < 0.001). These results suggest that the accuracy of width parameters was more reliably annotated than the accuracy of the height parameters, supporting the observation that height is more challenging to annotate consistently. Therefore, accuracy values in Table 4 need to be interpreted cautiously. Following the stricter annotation of the two raters, the number of outliers in the height parameter was reduced in the contrast videos (*n* = 197 in the raw videos, *n* = 157 in the contrast videos), and the number of outliers in the width parameter did not differ substantially between raw and contrast videos (*n* = 144 in the raw videos, *n* = 142 in the contrast videos). The contrast effect seemed to have improved the detection of Dlib’s central lower lip landmark (landmark 67). Therefore, the analysis of the vowel contrasts focused on the parameters measured by Dlib’s landmarks on the contrast videos.

**Table 4:** Descriptive results of the vowel measurements.

		Area		Height			Width		
V	*n*	*M*	*SD*	*M*	*SD*	acc.	*M*	*SD*	acc.
**/ɒː/**	52 (0.25)	708.6	87.92	19.231	2.348	13 (0.25)	56.38	2.143	13 (0.25)
**/ɛː/**	11 (0.05)	919.6	80.16	23.273	2.195	3 (0.27)	60.00	1.732	10 (0.91)
**/eː/**	28 (0.14)	886.7	97.88	22.714	2.401	1 (0.04)	59.54	2.380	25 (0.89)
**/iː/**	20 (0.1)	846.9	105.54	21.750	2.573	2 (0.1)	59.85	2.519	15 (0.75)
**/oː/**	25 (0.12)	461.6	94.65	13.120	2.386	11 (0.44)	55.88	3.283	0 (0)
**/uː/**	31 (0.15)	295.4	72.42	8.839	1.968	5 (0.16)	55.29	2.269	0 (0)
**/ʉː/**	21 (0.1)	349.0	70.55	9.524	1.601	8 (0.38)	59.14	2.414	0 (0)
**/yː/**	6 (0.03)	622.7	100.86	16.667	2.338	3 (0.5)	59.50	2.739	0 (0)
**/øː/**	11 (0.05)	747.4	107.36	20.182	3.157	2 (0.18)	56.55	3.446	0 (0)

The measured vowel categories (V) along with the absolute frequencies of the items (n, proportions in parentheses) and mean (M) and standard deviations (SDs) for the area, height, and width parameters in pixels. The accuracy rates (acc.) are presented as the absolute number of correctly located landmarks with proportion of accuracy in parentheses.

**Figure 8:**
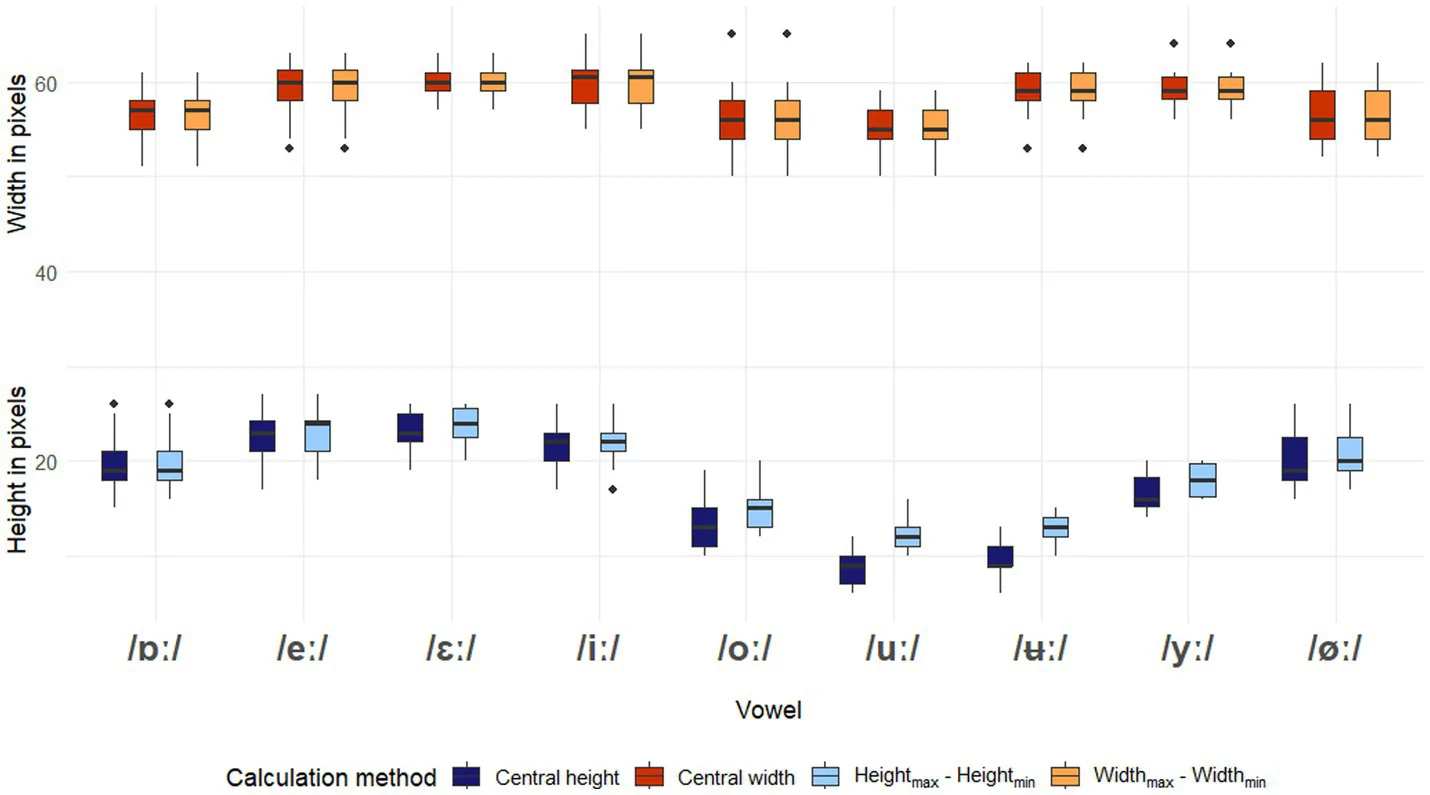
Boxplots comparing the subtraction method (lighter shades) and central measurements (darker shades) in Dlib’s output data. Height measurements are displayed in the bottom panel, width measurements are displayed in the lower panel.

Finally, Dlib’s central landmark calculations were checked in the contrast video data to explore whether they improved the accuracy of height and width measurements. Figure 8 shows the height values in pixels (lower panel) and width values in pixels (upper panel) for both calculation methods. As expected, the width measurements were not affected by the change of the calculation method. However, the height measurements changed to different degrees, and considerable changes occurred for the vowels /oː/, /uː/, /ʉː/, and /yː/. In other words, the vowels with narrower mouth openings showed the intended smaller height values after the central landmark calculations. Overall, the central problem seemed to be the algorithm’s limitation in detecting the corner of the mouth correctly. However, the improved height measurements provided support for choosing central landmark calculations over the subtraction method.

To summarize the evaluation process of the script, vowels measurements based on Dlib’s measurements of the contrast videos yielded the most accurate descriptions of the visual properties of the vowels. Dlib’s measures were more stable concerning the detection of a mouth opening, and the contrast videos seemed to support the algorithm in localizing the landmarks along the lips. Central landmark measurements were identified as the most accurate method concerning the height parameter, the width parameter was prone to inaccuracies in all conditions.

### 2.6 Data analysis (step 6)

Statistical analyses were performed on the continuous measures of area, height, and width in order to answer the research questions using the software R Studio (version 2022.02.2; R Core Team, 2022). The measures were not subject to any normalization procedure since all variables were measured on the same scale and all items were recorded with a consistent speaker-camera distance. Normality of the data distribution was tested with Shapiro–Wilk normality tests, separately for each parameter and vowel category. Differences across the vowel categories were tested for the area, height, and width parameters separately by employing nonparametric Kruskal-Wallis comparisons. Nonparametric tests were chosen because the samples are uneven and partially violated normality assumption, and a *post-hoc* Dunn’s test and Bonferroni corrections were applied to the outcome in order to adjust for multiple comparisons. The results are reported in section 3.

## 3 Results

Results are presented in the following order: First, we present a descriptive overview of the vowel measurements from the speaker of our case study. Second, we present the vowel contrasts that show significant differences in the measurements of the visual signal for the area, height, and width parameter separately (RQ_1_). Third, we present the results of the comparison with the phonological feature matrices (RQ_2_).

In Table 4, the results of the measurements are presented as an overview including the mean values and standard deviations for each vowel’s area, height, and width parameter. The full data is available at https://osf.io/qz4v9/. Close and rounded vowels (/oː/, /uː/, and /ʉː/) had a smaller average mouth opening area than the open and unrounded vowels (e.g., /ɛː/, /eː/, and /iː/). The data thus reflected the logical geometrical relation between one-dimensional length measures (i.e., height and width) and the two-dimensional area measure. The height measurements showed that the vertical mouth openings are largest for the vowel /ɛː/, /eː/ and /iː/, followed by /øː/ and /ɒː/. The width was larger for the open vowel /ɛː/, and for unrounded vowels (/eː/ and /iː/) than for /oː/, /uː/, /øː/ and even /ɒː/, suggesting that the vowel /ɒː/ could be rounded. Surprisingly, the data for vowel /ʉː/ fell outside of this pattern with width values closer to the width of /eː/ and /iː/. The same applied to the vowel /yː/, but this is a less surprising observation considering the type of lip rounding. The accuracy of the height and width parameter displayed in Table 4 is based on the manual annotation of two raters (first and third author), following the judgment of the stricter annotator.

### 3.1 Which Swedish vowel contrasts show significant differences in the visual signal in terms of area, height, and width of the mouth opening? (RQ_1_)

Shapiro–Wilk normality tests on the area, height, and width data revealed that the height data for the vowel /uː/ (*W* = 0.91594, *p* = 0.01845) and the width data for the vowel /eː/ (*W* = 0.90325, *p* = 0.01364) were not normally distributed. Table 5 shows the results of the nonparametric Kruskal-Wallis comparisons and *post-hoc* Dunn’s test (with Bonferroni-adjusted *p*-values). The tests identified 15 vowel contrasts with significant differences in the area of the mouth opening, 16 vowel contrasts with significant differences in the height of the mouth opening, and 12 vowel contrasts with significant differences in the width of the mouth opening (Table 5).

**Table 5:** Kruskal-Wallis comparisons with a *post-hoc* Dunn’s test of the area, height, and width measurements.

		Area				Height				Width			
/V1-V2/	Feature matrix	M_|V1-V2|_	*Z*	P.adj	*r*	M_|V1-V2|_	*Z*	P.adj	*r*	M_|V1-V2|_	*Z*	P.adj	*r*
/ɒː-ɛː /	Identical									3.62	−3.89758	**0.004**	0.491
/oː-ʉː/	Identical									3.26	−3.73501	**0.007**	0.551
/uː-ʉː/	Identical									3.85	−4.65181	**<0.001**	0.645
/oː-øː/	Identical					7.062	−3.2581	**0.040**	0.543				
/uː-øː/	Identical	−452	−5.0264	**<0.001**	0.776	11.34	−5.1532	**<0.001**	0.795				
/ʉː-øː/	Identical	398.4	−4.0940	**0.002**	0.724	10.66	−4.6160	**<0.001**	0.816				
/ɒː-eː/	Different	178.1	−3.7133	0.007	0.415	3.483	−3.2274	0.045	0.361	3.16	−4.86331	<0.001	0.544
/ɒː-iː/	Different									3.47	−4.38896	<0.001	0.517
/ɒː-oː/	Different	247	3.9664	0.003	0.452	6.111	4.0392	0.002	0.46				
/eː-oː/	Different	425.1	6.6715	<0.001	0.916	9.594	6.3219	<0.001	0.868	3.66	4.58741	<0.001	0.63
/ɛː-oː/	Different	458	5.4888	<0.001	0.915	10.15	5.0974	<0.001	0.85	4.12	3.91309	0.003	0.652
/iː-oː/	Different	385.3	5.5235	<0.001	0.823	8.63	5.1174	<0.001	0.763	3.97	4.25705	<0.001	0.635
/ɒː-uː/	Different	413.2	7.0415	<0.001	0.773	10.39	7.1073	<0.001	0.78				
/eː-uː/	Different	591.3	9.4670	<0.001	1.233	13.87	9.0875	<0.001	1.183	4.25	5.64378	<0.001	0.735
/ɛː-uː/	Different	624.2	7.4608	<0.001	1.151	14.43	7.0495	<0.001	1.088	4.71	4.63011	<0.001	0.714
/iː-uː/	Different	551.5	7.9828	<0.001	1.118	12.91	7.5483	<0.001	1.057	4.56	5.18207	<0.001	0.726
/ɒː-ʉː/	Different	359.6	5.2505	<0.001	0.615	9.707	5.8876	<0.001	0.689	2.76	−3.80300	0.005	0.445
/eː-ʉː/	Different	537.7	7.7178	<0.001	1.103	13.19	7.8939	<0.001	1.128				
/ɛː- ʉː/	Different	570.6	6.3895	<0.001	1.13	13.75	6.4040	<0.001	1.132				
/iː-ʉː/	Different	497.9	6.5589	<0.001	1.024	12.23	6.6394	<0.001	1.037				

The table shows the vowels in the contrast, whether the vowels in the contrast have identical or different feature matrices of roundedness and openness, along with the difference between the mean values (in pixels), the Z-score of the comparison, Bonferroni-adjusted *p*-values and effect size r. Boldface indicates significant comparisons that do not support the hypotheses.

While the majority of the contrasts in Table 5 were expected based on the underlying articulatory gestures, there was also a number of contrasts that were surprising, especially those in relation to the width parameter. For example, /ɒː/ as a presumed unrounded (or only *slightly* rounded) vowel differed significantly from the vowels /eː/, /ɛː /, and /iː/ in width, but not from, for example, the vowels /oː/, and /øː/, suggesting more similarities to rounded vowels. It was also striking that none of the vowels differed significantly from /yː/, in either of the visual parameters. A further unexpected finding was that /ʉː/ did not differ in width from the unrounded vowels /eː/, /ɛː/, and /iː/, but instead differed from /uː/ in width. However, as discussed in 4.3, contrasts involving /ʉː/ must be interpreted carefully due to measurement errors in the width parameter. For the same reason, there also was a strong tendency for vowel contrasts that differed significantly in height to also differ significantly in terms of area.

### 3.2 Do the binary phonological descriptions of vowel roundedness and openness match the visual lip configurations of Swedish vowels? (RQ_2_)

We hypothesized that vowel contrasts with identical feature matrices for roundedness and openness should not show significant differences in the visual parameters area, height, and width, that is, they should look the same. The results supported the hypothesis for the contrasts /eː–iː/, /ʉː–yː/, /yː–øː/, /uː–yː/, /oː–yː/, and /oː–uː/. However, visual differences were found for /ɒː–ɛː/, /oː–ʉː/, /uː–ʉː/, /oː–øː/, /uː–øː/, /ʉː–øː/, although these have identical feature matrices (Table 5). Strikingly then, half of the vowel contrasts with identical features matrices showed visual differences that could not be predicted by the binary description of openness and roundedness.

We also hypothesized that vowel contrasts with differences in feature matrices for roundedness and openness should show significant differences in at least one of the visual parameters area, height, and width, that is, they should look different. Support for this hypothesis was found in 14 of the 24 contrasts in either one or more of the three parameters (Table 5). However, 10 contrasts did *not* show significant differences in any of the parameters (/eː–ɛː/, /ɛː–iː/, /ɒː–yː/, /eː–yː/, /ɛː–yː/, /iː–yː/, /ɒː–øː/, /eː–øː/, /ɛː–øː/, /iː–øː/). In other words, almost half of the vowel contrasts with different feature matrices did not show visual differences predicted by the binary description of openness and roundedness.

**Figure 9:**
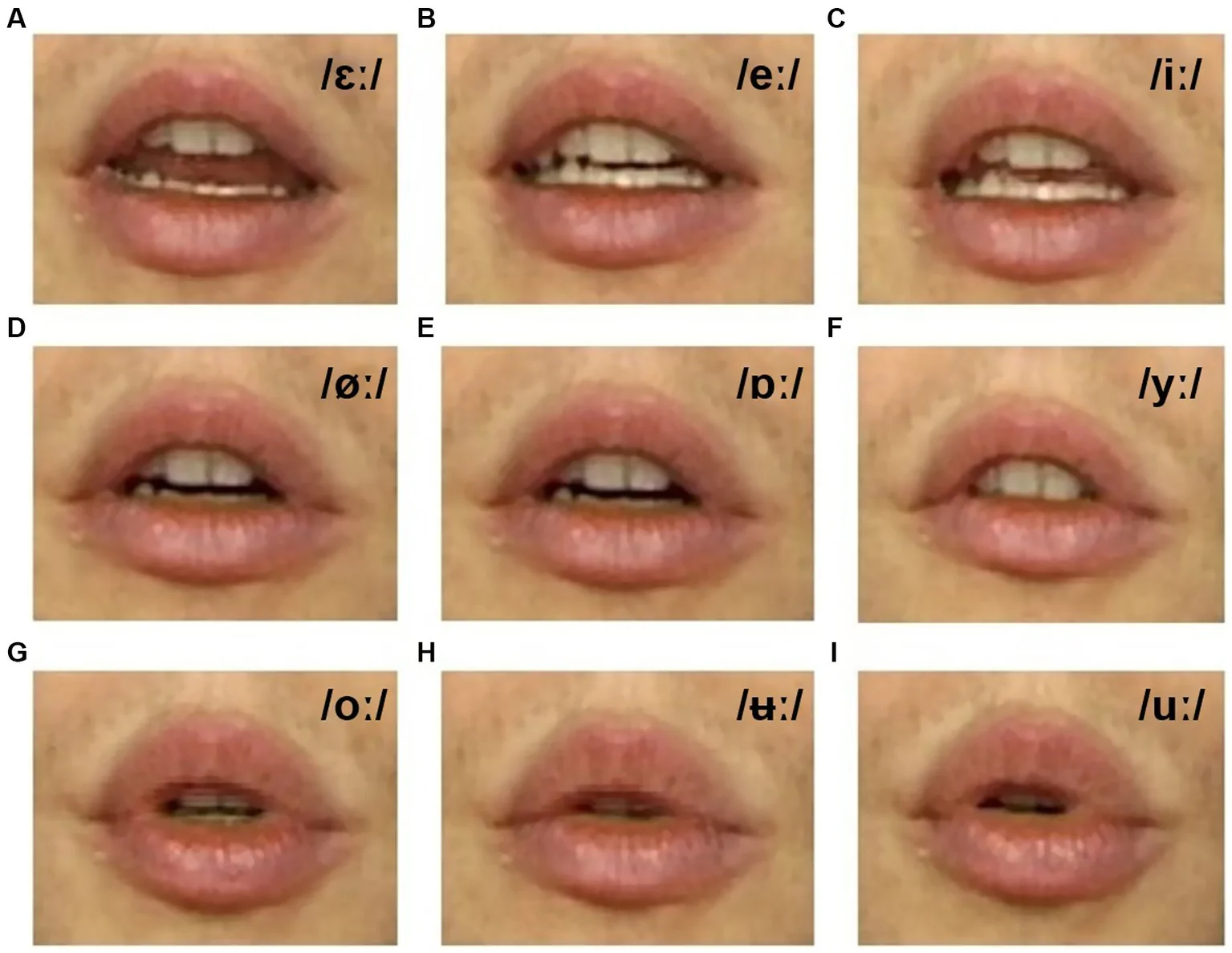
Lip configurations of the nine long vowels in Swedish, presented in descending order according to the area of the mouth opening [/ɛː/ **(A)**, /eː/ **(B)**, /iː/ **(C)**, /øː/ **(D)**, /ɒː/ **(E)**, /yː/ **(F)**, /oː/ **(G)**, /ʉː/ **(H)**, and /uː/ **(I)**].

As displayed in Figure 9, the lip configurations of the nine long vowels range from wide open to extremely narrow mouth openings. This gradual pattern from the current study’s measurements may roughly correspond to the feature matrices, but also shows deviations from the feature-based predictions.

## 4 Discussion

This study provided measurements of the visual characteristics of the mouth opening and lip configurations of long vowels in Swedish, examining (1) which Swedish vowel contrasts show significant differences in the visual signal (in area, height, and width) in continuous speech, and (2) whether phonological feature descriptions sufficiently predict the visual properties of vowels as they can potentially be available to the addressee. The results showed that there are gradual visual differences within and between the measured nine long vowel categories, and that the visual parameters formed vowel clusters of visually similar lip configurations. Although statistical significance in this context cannot be equated with perceptibility, it provides an objective cutoff point for potentially noticeable differences in the signal. Vowel contrasts involving significant visual differences can thus be considered high-salient contrasts. Furthermore, the results showed that six of the 12 vowel contrasts expected to look the same based on feature matrices showed visual differences, and conversely, that 10 of the 24 vowel contrasts showed no significant visual differences despite being described by different feature matrices. The results thus suggest that visual contrasts in continuous speech are gradual and do not entirely correspond to the binary feature matrices of openness and roundedness (Table 2). Consequently, determining visual salience of vowel contrasts based on phonological feature matrices risks being misleading. In the remainder of section 4, we discuss the results in relation to visually salient contrasts of Swedish in previous studies, the drawbacks of a phonological feature description of visual salience, and the computer vision approach, respectively.

### 4.1 Visually salient vowel contrasts in continuous speech

An inherent aspect of continuous speech is the variation in the realization of vowels and consonants due to coarticulation. In contrast to the majority of studies on visual lip configurations of vowels, the current study did not limit the vowel items to a specific coarticulatory context, thereby capturing the linguistic reality of the visual speech signal. Importantly, despite the coarticulatory variation of the target vowels, there are general patterns emerging from the data (i.e., clusters of vowels) suggesting that visual differences between lip configurations appear even in continuous speech. The patterns for the vowels /ɒː/, /eː/, /iː/, /oː/, /uː/, and /ʉː/ are based on a solid sample of vowel tokens in a wide range of coarticulatory contexts but given the small sample size for the vowels /ɛː/, /yː/, and /øː/, these must be treated with greater caution.

The patterns in the area and height measurements are similar to patterns from the visual measurements of Swedish vowels provided by Ericsdotter (2005), suggesting that the overall patterns of visual parameters of vowels in continuous speech generally align with measurements made from sustained vowels or isolated words. This could be assumed to hold for the width measurements as well. However, the current study was not able to provide highly accurate width measurements (cf. 4.3). Therefore, the results of the statistical analysis for the width parameter are included mainly as a methodological illustration. In their current form, they are not reliably capturing the width of the inner mouth opening and need to be interpreted with caution.

Although differences in the visual parameters of vowels cannot be directly transferred to speechreading performances, measurements of lip configurations can point toward pairs of vowels that could potentially be challenging to addressees. The patterns emerging from the area and height data are generally in line with Amcoff’s (1970) visually contrastive groups (/iː, eː, ɛː/, /ɒː, øː, yː/, and /oː, ʉː, uː/). For example, although the visual measurements of the vowel category /oː/ indicate slight visual differences between /oː/ compared to /ʉː/ and /uː/, the lack of significance between their visual parameters, and the perceptual performance in Amcoff’s (1970) speechreading participants suggest that the visual differences may not be strong enough to enable good visual-only discrimination. Moreover, as discussed in Mártony’s (1974) speechreading study, such perceptual performance also depends on the speaker pronouncing the target items.

While sustained vowels or monosyllabic stimuli are preferred in many experimental contexts, the chosen material from continuous speech reflects the everyday linguistic input of listeners and is similar to material used in many audiovisual processing studies. The current study is a proof-of-concept case study and does not account for anatomical differences and individual speaking styles, which could add more variety to the patterns. Future studies should replicate the current measurements with a larger number of speakers and an evenly distributed set of vowel tokens per category to test whether the present data (more specifically, the relations between the vowel categories, not the absolute values) hold for a larger group of speakers and measured tokens. Moreover, other potentially informative parameters such as movements in jaw and cheeks may be included in future studies, as well as the protrusion parameter from a lateral view. However, the question remains whether these descriptions are better suited than phonological features for the prediction of perceptual salience of audiovisual vowel contrasts. The current study includes the complete set of long vowels in Swedish and can complement and add insights to previously conducted measurements of Swedish vowels in different settings using different elicitation methods.

### 4.2 Describing visual differences: gradient parameters or categorical features?

The first hypothesis predicted that vowel contrasts with identical feature matrices of roundedness and openness should not show significant differences in the visual parameters area, height, and width. This was not supported by the data. Half of the pairs with identical phonological feature matrices showed visual differences that could not be accounted for solely by binary features (i.e., the matrices specified in Table 2). One possible reason for this is that phonological features describe the tongue’s position in the mouth, not the parameters of the mouth opening that are concomitant with articulatory tongue gestures. For example, the height of the mouth opening available for the addressee cannot be predicted straightforwardly by using the ±open feature, as shown by the data for the vowels /eː/ and /iː/, characterized as close vowels (i.e., the tongue being closer to the palate) yet being two of the vowel categories with the largest vertical mouth opening, partly due to the increased spread of the lips (Figure 9, upper panel). In other words, the area and height parameters do not correspond 1:1 to the openness feature, and it is therefore not sufficient to predict visual differences between vowels. This observation may not be novel, but it underscores the importance of quantified descriptions of the visual signal.

A more unexpected finding was that the vowel /ɒː/ seemed to be closer in width to the rounded vowels, which may indicate rounding or may be a byproduct of the number of items in this category. If lip rounding is present for /ɒː/ in the audiovisual speech signal, this is not in line with traditional descriptions of this vowel category in Swedish (Engstrand, 1999; Riad, 2013). It will be important in future studies to determine whether this observation holds for a larger set of speakers or whether this finding is speaker-specific. The current data also suggest visual differences in height and width between the vowels classified as +rounded. Therefore, the width of the mouth opening does not seem to correspond 1:1 to a binary roundedness feature. The vowels /uː/ and /ʉː/ were produced with a compressed rounding, which means that their mouth opening should be narrower compared to that of the other rounded vowels /øː/, /yː/, and /oː/, produced with protruded lips. Although the width differences in our data may be partially caused by an inaccurate placement of landmarks (e.g., /oː–ʉː/ and /uː–ʉː/) (Table 5), the visual inspection of the lip configurations suggests that there could be visual differences separating /øː/, /yː/, and /oː/ from /uː/ and /ʉː/ (Figure 9, lower panel).

The second hypothesis, stating that different phonological feature matrices should lead to visual differences, was also not borne out. The data showed no such differences for 10 of the vowel contrasts. One example is the vowel /yː/, which did not contrast significantly with any other vowel, which is unlikely given the descriptive data. This could have been caused by the small sample sizes of /yː/, /ɛː/ and /øː/ and the landmark inaccuracy. However, it could also be the case that the width difference is not visually contrastive in /ɒː–yː/, /eː–yː/, /ɛː–yː/, /iː–yː/, /ɒː–øː/, /eː–øː/, /ɛː–øː/, and /iː–øː/ because the protruded rounding does not lead to the same height and width reduction as compressed rounding does. Moreover, the findings point toward visual similarities between /ɛː/, /eː/ and /iː/. Both these data and the speechreading results of Amcoff (1970) are therefore at odds with predictions made by simple binary phonological feature matrices. These findings will need to be confirmed in larger and more balanced samples. However, for now it seems fair to assume that there are more similarities between, for example, /iː/ and /yː/ than previously assumed. If the protruded lip rounding in Swedish disappears perceptually and in production, the phonological feature +rounded is misleading, especially in speakers who show stronger tendencies toward the merged form, or in those who have in-between forms (Gross and Forsberg, 2020). A further question arising is whether the visual differences are caused by lip protrusion and thus predominantly reside in the lateral perspective. It remains for further research to determine whether lateral visual differences are significant in continuous speech as well, or whether they diminish under such conditions.

If the vowel lip configurations are described based on phonological binary features, languages such as Swedish require at least one more feature to account for the visual differences between protruded and compressed rounding. However, even such an approach risks neglecting gradual differences that might be visible to the addressee (e.g., comparing /øː/, /yː/, and /oː/, and /ɒː/). In other words, the binary phonological feature descriptions of openness and roundedness do not match the visual lip configurations of long Swedish vowels particularly well. The quantitative approach exemplified here takes more into account than a discrete description of tongue position and lips and focuses on quantified visual parameters that may be available in human speech perception. In this way, we can account for different types of rounding, but also provide gradual reference material to describe the signal which may be processed using gradual information instead of, or complementary to, discrete features.

However, in perception studies assessing audiovisual speech benefits, it remains uncommon to account for the visual salience of speech signals based on quantified, listener-independent measurements. While the use of feature-based descriptions may be sufficient for studies focusing on a small number of specific contrasts, these risk oversimplifying or misrepresenting differences in the salience of vowel contrasts when the whole vowel system is examined. Therefore, we argue for the use of quantified and listener-independent parameters to describe the visual salience of the speech signal, which can subsequently be used to examine potential perceptual effects, that is the perceptual salience of these contrasts. Despite human listeners’ remarkable visual perception skills, it remains open whether they can detect fine-grained visual differences and how their capacity for such detection compares to computer-vision-based analyses, especially given the high variability and speed of the incoming signal in natural speech. Importantly, computer-vision-based descriptions cannot make any statements about perceptual salience, only quantify potentially available information, whose relation to perceptual salience is yet to be established in future studies.

### 4.3 Measuring lip configurations with computer-vision-based facial landmarks

The current study reveals the potential of a computer-vision-based method for extracting landmarks on the face for examining two-dimensional characteristics of vowel lip configurations. The overall patterns of area, height, and width measurements align with what the addressee can see in the visual signal (Figure 9). The visual area parameter was retrieved from the video frames by adding up all pixels within the outline of the polygon. Provided that the landmarks are detected correctly, this method seems very useful. The central landmark method also proved to be promising for the calculation of the height and width of the mouth opening. The script executed the many steps of processing the video material to the final result of area, height, and width measurements in an efficient manner, and is thus a versatile and efficient tool for examining the visual parameters of speech.

However, the computer vision approach comes with several challenges, general as well as specific to the issue of landmark accuracy. According to Brooke and Summerfield (1983), measuring articulatory movements in the face means overcoming three major challenges: defining a reference frame of the axis of the measurements, distinguishing facial articulatory movements from those of the head or torso, and achieving the degree of precision required due to the relatively small dimensions of articulatory movements of the mouth. Facial landmark tracking has become more accurate recently and now allows for a more reliable detection of facial landmarks on the mouth despite slight head movements (Lee et al., 2019). However, a fixed head position is an advantage especially in measurements without any markers specifying a reference frame, for example in the form of sensors, colors, glasses, helmets, metal wires or bite plates. Although the speaker in our materials was at a constant distance from the camera in all recordings, future studies of this kind must include reference points to ensure with higher certainty that the measurements are not affected by head movements. However, eliciting speech without fixing the head’s position has the advantage of ensuring natural articulatory gestures. Head-mounted cameras in combination with facial landmark extraction are one of the suggested solutions to this problem, allowing for free head movement and reliable extraction of visual lip parameters (Krause et al., 2020). Future work may also include facial reference distances for normalization and conversion of the pixel-based measurements into physical units, which is needed especially when multiple speakers and camera-speaker distances are involved.

A related key issue is how to measure vowel midpoints. First, the minor shifts of the measured frames in relation to the annotated time stamps of a maximum of 39 milliseconds could have shifted the measured frames slightly from the determined midpoints. However, considering that the average duration of a Swedish long vowel in sentences is approximately 158 milliseconds (Elert, 1964), this should not have affected the measurements. Second, diphthongization is a process that can affect almost all long vowels in Swedish (Riad, 2013) and that could change the lip configuration during the articulation of a long vowel. However, there were no indications of strong effects of diphthongization on the visual signal recognizable during the annotation.

The central challenge of the method in the current study is the accuracy of the facial landmark placement. In this methodological procedure, we tested two libraries, Dlib and MediaPipe. In Roesler et al. (2022), MediaPipe yielded more accurate landmark predictions than Dlib for four landmarks on the *outer* outline of the lips in four speech tasks. The current data suggest that both libraries are sensitive to lighting conditions and prone to error in the detection of the landmarks on the corners of the mouth along the *inner* mouth outline. Although our data show some gradual changes in width, it is likely that rounded vowels pose a problem for these algorithms (Figures 4, 5, 7). It seems improbable that this is solely caused by shadows misleading the algorithm based on lighting conditions since the oral cavity and the teeth also provide strong contrasts which should aid in landmark detection. It seems more likely that rounding the lips smooths the edge of the oral commissures, which turns an acute angle into a curved line. This might be a less frequent lip configuration in the training material of these algorithms.

As a consequence of the landmark misplacement, there was less gradual variation in the width data. For example, both /yː/ and /ʉː/ had remarkably high width values, although lip rounding is expected to lead to a smaller width of the mouth opening, especially for /ʉː/ which is produced with a compressed rounding and thus a very narrow mouth opening. In protruded rounding, a larger mouth opening can be retained, which can cause the larger width for the vowel /yː/ (Lindau, 1978). The accuracy rates (Table 4) suggest that the rounding of the lips, and the resulting narrower horizontal opening, led to inaccuracies in the landmark detection on the corners of the mouth. These landmarks were detected inaccurately for all items of the categories /oː/, /uː/, /ʉː/, /yː/, and /øː/, and for 75% of the items measured for /ɒː/. This tendency remained even in the contrast videos. In other words, although some of the width data are in line with what would be expected based on the articulation of the vowels, they need to be interpreted with caution, since the actual mouth opening for the rounded vowels is smaller than the width output that Dlib’s algorithm generates in our script (see Figure 9, lower panel).

Inaccurate landmark detections also affected the height data in all vowel categories (Table 4). Even after adjusting the picture contrast, the algorithm still misplaced the landmark at the light reflection on the lower lip instead of its actual boundary to the mouth (Figures 6, 7). Therefore, the absolute values of the height data need to be interpreted cautiously. However, the relative differences between the height of the different vowel categories have been preserved despite the landmark error. In other words, there was a distortion of the values, but it was systematic and appeared in all vowel categories. We conclude that lighting conditions are crucial for the accuracy of facial landmark detection. The quality of the video material may have also affected the measurements. Future studies with material of higher resolution and careful lighting promise higher precision in determining the visual parameters of the mouth opening. Machine lipreading systems nowadays already achieve high accuracy, and insights from the development of these applications and systems may be used in future studies for improving research tools for speech measurements.

The data generated with the current method is thus in need of post-processing of the landmarks along the inner mouth outline to achieve higher accuracy. Quantifying lip configurations from video frames (i.e., extracting area, height, and width parameters) requires a more precise landmark localization, and/or an approach including reference markers or color on the lips. However, facial landmark detection still proved to be a promising starting point to measure the visual lip configurations of speech sounds. Alternatively, future work may use segmentation-based approaches to represent the mouth opening more accurately.

In conclusion, this proof-of-concept study examined visual salience of speech sounds by quantifying lip configurations in continuous speech to characterize what visual information is available to listeners. The results showed that computer vision based geometric lip configurations reveal visually distinguishable groups of Swedish long vowels in continuous speech. Moreover, traditional production-based binary features can misrepresent the visual information that is available to addressees. As increasingly acknowledged, visual lip configurations of vowels are as variable as their acoustic counterparts in audiovisual speech. A quantified description of the visual signal therefore offers a complementary and more fine-grained description of the visual cues available in audiovisual communication than traditional feature-based descriptions. If visual salience is required to gain a perceptual benefit from audiovisual speech, it is crucial to determine how it can be measured to improve our understanding of its potential effects in perception. Contrasts that are assumed to be visually different on theoretical grounds may show no significant visual difference in continuous speech, leading to a lack of facilitatory effects. The current study provides a baseline for separating high- from low-salient visual contrasts, as compared to feature-based baselines. Subsequent studies will show whether these quantified measures of visual salience reflect perceptual salience and can predict facilitatory effects of audiovisual speech. These realizations have implications for studies of speech synthesis and facial animation, as well as for studies of speech processing and language acquisition. This study has suggested a way forward for improving our understanding of the salience of speech sounds in the audiovisual signal from more naturalistic speech material.

## Data Availability

The datasets presented in this study can be found in online repositories. The names of the repository/repositories and accession number(s) can be found at: https://osf.io/qz4v9/.

## Footnotes

1 We use the symbol /ɒː/ to indicate that this vowel can be slightly rounded in Central Swedish. However, in binary feature approaches, it is not typically classified as a rounded vowel (Engstrand, 1999; Riad, 2013).

2
https://osf.io/qz4v9/
